# Validation of Serum Biomarkers That Complement CA19-9 in Detecting Early Pancreatic Cancer Using Electrochemiluminescent-Based Multiplex Immunoassays

**DOI:** 10.3390/biomedicines9121897

**Published:** 2021-12-14

**Authors:** Jin Song, Lori J. Sokoll, Daniel W. Chan, Zhen Zhang

**Affiliations:** 1 Center for Biomarker Discovery and Translation, Department of Pathology, Johns Hopkins University School of Medicine, 419 North Caroline Street, Baltimore, MD 21231, USA; lsokoll@jhmi.edu (L.J.S.); dchan@jhmi.edu (D.W.C.); 2 The Sidney Kimmel Comprehensive Cancer Center, Department of Oncology, Johns Hopkins University School of Medicine, Baltimore, MD 21287, USA

**Keywords:** multiplex, immunoassay, serum, biomarker, pancreatic cancer

## Abstract

Pancreatic ductal adenocarcinoma (PDAC) is a lethal malignancy; its early detection is critical for improving prognosis. Electrochemiluminescent-based multiplex immunoassays were developed with high analytical performance. All proteins were analyzed in sera of patients diagnosed with PDAC (*n* = 138), benign pancreatic conditions (111), and healthy controls (70). The clinical performance of these markers was evaluated individually or in combination for their complementarity to CA19-9 in detecting early PDAC. Logistic regression modeling including sex and age as cofactors identified a two-marker panel of CA19-9 and CA-125 that significantly improved the performance of CA19-9 alone in discriminating PDAC (AUC: 0.857 vs. 0.766), as well as early stage PDAC (0.805 vs. 0.702) from intraductal papillary mucinous neoplasm (IPMN). At a fixed specificity of 80%, the panel significantly improved sensitivities (78% vs. 41% or 72% vs. 59%). A two-marker panel of HE4 and CEA significantly outperformed CA19-9 in separating IPMN from chronic pancreatitis (0.841 vs. 0.501). The biomarker panels evaluated by assays demonstrated potential complementarity to CA19-9 in detecting early PDAC, warranting additional clinical validation to determine their role in the early detection of pancreatic cancer.

## 1. Introduction

Pancreatic ductal adenocarcinoma (PDAC) is a lethal malignant tumor with high metastatic potential. Most patients are diagnosed at advanced stages, with a median survival of 6 months and an overall 5-year survival of <5% [1]. The early detection of PDAC is critical because surgery at an early stage is the most promising therapy that greatly improves prognosis [2]. However, there are currently no sufficiently sensitive or specific screening tests for the early detection of PDAC. Conventional imaging tools, including abdominal computerized tomography (CT) scanning, magnetic resonance imaging (MRI), endoscopic retrograde cholangiopancreatography (ERCP) and endoscopic ultrasound (EUS) are inadequate for detecting small premalignant lesions and are relatively costly, time-consuming and invasive [3]. The current gold-standard serum marker CA19-9 is used in the clinic only for disease monitoring, because it lacks the necessary sensitivity and specificity due to its absence in 5–10% of patients with a Lewis-negative genotype and because it is frequently elevated in non-malignant conditions, such as pancreatitis and other benign conditions [4,5,6]. All of these factors limit its clinical utility in a screening and early detection setting. There is an urgent clinical need to identify additional biomarkers to complement CA19-9 for the early detection of PDAC.

Multiplex immunoassays simultaneously measure multiple analytes in a single sample, providing quantitative data via parallel analyses, which is especially suitable for serum biomarker verification and validation. The multiplex immunoassay platforms confer several advantages over traditional enzyme-linked immunosorbent assays (ELISAs), such as increasing productivity, conserving critical reagents and samples, and delivering results quickly. The simultaneous analysis of multiple biomarkers makes it possible to identify combinations of biomarkers that have greater disease specificity and sensitivity than results obtained from the analysis of any single marker. Compared with different commercial multiplex immunoassay platforms, including both plannar array and microbead assays, Meso Scale Discovery (MSD)’s MULTI-ARRAY system and Bio-Rad’s Bio-Plex system using Luminex xMAP technology were found to have the best performance with the lowest limits of detection, and the MULTI-ARRAY system had the greatest linear signal output over the widest concentration range (10^5^ to 10^6^) [7,8]. Previously, we successfully developed magnetic bead-based multiplex immunoassays of serum biomarkers using a Bio-Plex 200 suspension array system (Bio-Rad, Hercules, CA, USA) [9,10,11,12], and applied these markers to a case-control set of serum samples from subjects with PDAC or benign conditions, and healthy controls [11]. In this study, electrochemiluminescent-based multiplex immunoassays were developed for additional selected serum biomarkers using a MESO QuickPlex SQ 120 instrument (MSD, Rockville, MD, USA), and applied to a collection of patient serum samples. The performance of these candidate biomarkers was evaluated individually and in combination for their ability to complement CA19-9 for the early detection of PDAC.

## 2. Materials and Methods

### 2.1. Specimens

A total of 319 archived serum samples obtained from 138 patients with histologically diagnosed PDAC, 111 patients with benign pancreatic conditions, including both intraductal papillary mucinous neoplasms (IPMN) and chronic pancreatitis (CP), and 70 healthy controls without a history of pancreatic diseases were studied with institutional approval. All patient serum samples were obtained before surgery or other treatment, and stored at −80 °C until analysis.

### 2.2. Reagents and Antibodies

The selected serum biomarkers for the development of electrochemiluminescent-based multiplex immunoassays included cancer antigen 125 (CA-125), human epididymis protein 4 (HE4), cytokeratin-19 (KRT19), folate receptor 1 (FOLR1), carcinoembryonic antigen (CEA), hepatocyte growth factor (HGF), osteoprotegerin (OPG), and TEK receptor tyrosine kinase (Tie-2). The recombinant proteins and antibodies used for the development of the first 4-plex assay were purchased from the following commercial sources: recombinant human CA125/MUC16 (5609-MU-050), recombinant human FOLR1 protein (5646-FR-050), human FOLR1 antibodies (MAB5646 and AF5646), and human cytokeratin 19 antibody (MAB3506) were from R&D Systems (Minneapolis, MN, USA); CK19 recombinant protein (MBS355584) and mouse CA125 monoclonal antibodies (MBS568141 and MBS568096) were from MyBioSource (San Diego, CA, USA); mouse HE4 monoclonal antibodies (3C24 and 2B13) were from Advanced ImmunoChemical (Long Beach, CA, USA); recombinant human HE4 (230-3001-10) from RayBiotech (Norcross, GA, USA); and mouse Cyfra-21-1 antibody (ABIN573309) from Antibody-Online (Atlanta, GA, USA). Antibodies of MBS568141, 3C24, ABIN573309 and MAB5646 or MBS568096, 2B13, MAB3506 and AF5646 were used as capture antibodies or detection antibodies in their respective assays. The R-PLEX antibody sets of CEA (F21QE-3), HGF (F214N-3), OPG (F21ZK-3) and Tie-2 (F210T-3) used for the development of the second 4-plex assay were purchased from MSD (Rockville, MD, USA). MSD GOLD SULFO-TAG NHS-Ester Conjugation Pack 1, GOLD 96-well small spot streptavidin SECTOR plate, U-PLEX Development Pack (4-Assay, K15229N-4), MSD Blocker A Kit, Wash Buffer (20×), MSD Read Buffer T (4×), and GOLD Read Buffer (1×) were also purchased from MSD (Rockville, MD, USA). EZ-Link^TM^ Sulfo-NHS-LC-Biotin and Zeba^TM^ spin desalting columns were purchased from Thermo Scientific (Rockford, IL, USA). Serum CA19-9 concentrations were measured using an FDA-cleared assay on the Tosoh AIA-600II immunoassay analyser (Tosoh Bioscience, San Francisco, CA, USA). In a subset of patient samples, serum CA-125 concentrations were also measured using an FDA cleared assay on the Tosoh AIA-2000 immunoassay analyser (Tosoh Bioscience, San Francisco, CA, USA).

### 2.3. Biotinylation and SULFO-TAG Conjugation of Antibodies

Capture and detection antibodies for CA-125, HE4, KRT19 and FOLR1 were either biotinylated using EZ-Link^TM^ Sulfo-NHS-LC-Biotin or SULFO-TAG conjugated using MSD GOLD SULFO-TAG NHS-Ester Conjugation Pack 1, according to the manufacturer’s instructions. The optimal challenge ratios for biotinylation and SULFO-TAG conjugation were either 50:1 or 20:1. The biotinylated capture antibodies and SULFO-TAG-conjugated detection antibodies were purified using Zeba^TM^ spin desalting columns and stored in storage buffer provided in the kit at −80 °C in the dark. The R-PLEX antibody sets include a matched biotinylated capture and SULFO-TAG conjugated detection antibody pair and a calibrator for the quick development of immunoassays on MSD plates.

### 2.4. Multiplex Immunoassay

**Figure 1:**
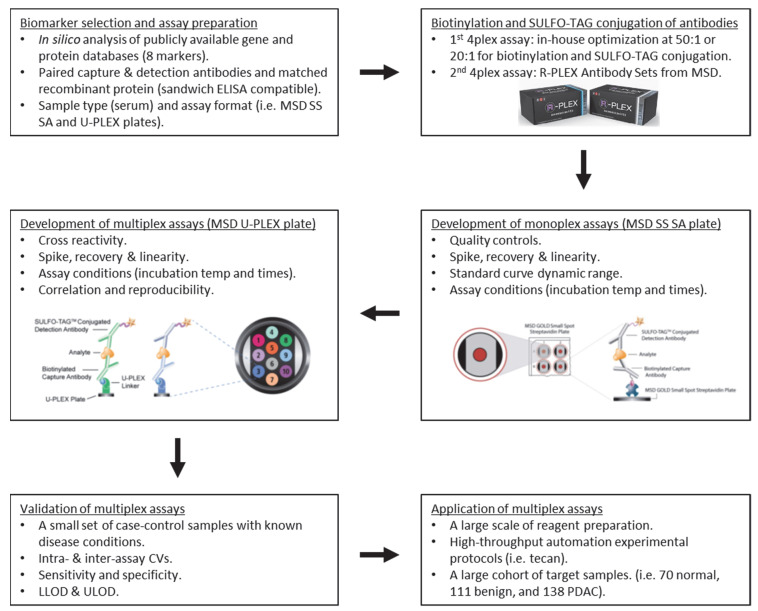
Flowchart of the development and application of two 4plex MSD assays.

Electrochemiluminescent-based multiplex immunoassays were developed for the selected candidate serum biomarkers using a MESO QuickPlex SQ 120 instrument (MSD, Rockville, MD, USA). The flowchart of the development and application of MSD multiplex immunoassays is shown in Figure 1. The monoplex immunoassays for individual candidates were first developed on MSD GOLD small spot streptavidin (SS SA) plates. Briefly, the streptavidin plates were blocked with 150 µL/well of MSD Blocker A solution with shaking for 1 h. After washing 3 times with 200 µL/well of 1× MSD wash buffer, the plates were coated with 25 µL/well of biotinylated capture antibody overnight at 4 °C. After washing 3 times, the plates were incubated with 50 µL/well of diluted calibrators or controls with shaking for 2 h. After washing 3 times, the plates were incubated with 25 µL/well of SULFO-TAG conjugated detection antibody with shaking for 1 h. The plates were finally washed 3 times, after which 150 µL/well of 1× read buffer was added, and read on an MSD instrument. Three pooled human sera with known protein measurements, two internal quality controls (QCs) at either high or low levels and one Sigma QC (S7023; Sigma-Aldrich, Inc., St. Louis, MO, USA), were used as the controls to optimize the assay conditions.

Before multiplexing the individual assays, assay specificity was examined by performing single-antigen and single-detection antibody cross-reactivity studies to detect the fluorescence signals in response to high concentrations of the recombinant proteins minimally at the 3rd dilution point of the standard curve. The single antigen study was conducted by testing an individual antigen in the presence of multiplexed capture and detection antibodies, which evaluates the specificity of a capture antibody. The single detection antibody study was conducted by testing an individual detection antibody in the presence of multiplexed capture antibodies and antigens, which evaluates the specificity of a detection antibody and, to some degree, the specificity of the capture antibody. Cross-reactivity was defined as the percentage of nonspecific cross-reacting signal detected relative to the specific signal for that analyte.

Two 4-plex assays were developed using a U-PLEX Development Pack (4-Assay) on the MSD instrument. For the multiplex immunoassay, individual U-PLEX-coupled antibody solutions at 10× the coating concentration were first created by coupling an individual biotinylated capture antibody to a unique linker, then multiplex coating solution was prepared by combining equal volume of each U-PLEX-coupled antibody solution and bringing the solution up with stop solution to result in a final 1× coating concentration at 1 µg/mL. The final concentrations of the SULFO-TAG conjugated detection antibodies in the multiplex assay were used at 1 µg/mL for CA-125, 0.125 µg/mL for HE4, 2 µg/mL for KRT19 and FOLR1, and 0.1 µg/mL for CEA, HGF, OPG and Tie-2, respectively, after titration. Calibration curves were established using either 11 or 7 calibrators in a 4-fold dilution series in the standard diluent derived from a mixture of the highest standard points of 4 recombinant proteins. The highest standards for the recombinant proteins in two 4-plex assays were 500, 30, 600 and 700 ng/mL for CA-125, HE4, KRT19 and FOLR1 (1st 4-plex), and 50, 12, 100 and 40 ng/mL for CEA, HGF, OPG and Tie-2 (2nd 4-plex), respectively. The multiplex immunoassays were compared to the monoplex immunoassays for protein quantifications of the candidate proteins in 30 patient sera. The correlation of the developed multiplex immunoassay and the Tosoh Bioscience AIA-2000 assay for serum CA-125 protein quantification was also determined in 30 patient sera. The multiplex immunoassay was carried out with the same procedures as those in the monoplex assays described above, except that the coating of U-PLEX plates was followed by blocking plates, using 50 µL/well for all incubation steps of antibody and antigen, and 2× read buffer was used for reading plates. The serum samples were either 4- or 8-fold diluted in the sample diluent in the multiplex immunoassays. The multiplex immunoassays were performed in duplicate on 96-well U-PLEX plates. All samples were randomized with regard to their plate locations. All assays were carried out at room temperature and protected from light. High-throughput automation experimental protocols were established. Tecan Freedom EVO 100 platform (Tecan US Group Inc., Morrisville, NC, USA) was used for the samples dilution and dispensing. All wash steps were performed with the wash buffer on an automated plate washer (BioTek ELx50 Microplate Strip Washer, Winooski, VT, USA).

Data acquisition and primary data analysis were performed on a MESO QuickPlex SQ 120 instrument in combination with Discovery Workbench 4.0 by use of a 4-parametric (4-PL) nonlinear logistic regression curve fitting model (MSD, Rockville, MD, USA). Assay analytical sensitivity (lower limit of detection, LLOD) was defined as the calculated concentration of analyte corresponding to the signal 2.5× standard deviations (SD) above the background (zero calibrator). The upper limit of detection (ULOD) was defined as the calculated concentration of analyte corresponding to the signal 2.5× SD below the upper plateau of the standard curve. HillSlope was determined from the curve fit. Intra-assay precision was calculated as the coefficient of variance (%CV) for at least 46 replicates of the pooled human sera within a single assay. Inter-assay precision was calculated as the %CV from at least 9 independent assays. The assay recovery was calculated as the percentage of the observed concentration relative to the expected concentration of each standard point and the spiked calibrators at low and high levels. The assay working dynamic range was defined as the range between LLOD and ULOD, for which the assay was both precise (intra-assay %CV ≤10% and inter-assay %CV ≤15%) and accurate (80–120% recovery).

### 2.5. Data Analysis

Biomarker data were transformed prior to analysis (log-transformation followed by z-score). Analysis of variance (ANOVA) and the nonparametric Mann–Whitney U test were used to compare serum biomarker levels between subjects with PDAC and benign pancreatic conditions or healthy controls, with a *p*-value less than 0.05 considered significant. Logistic regression modeling was constructed including sex and age as cofactors and the backward stepwise selected z-score transformed variables with the highest performance. Receiver-operating-characteristic (ROC) curve analysis was performed and the area under the curve (AUC) was calculated separately for individual biomarkers and the combinations of biomarkers. The Delong test was used to compare the AUCs. For the identified multivariate panels, the improvement in sensitivity (SN) at a fixed level of specificity (SP) was further assessed. Pearson correlation coefficients were determined to assess the correlation of the measurements between the multiplex and monoplex immunoassays or commercial kit, and were also used to evaluate the association of markers with age and gender separately in the healthy controls, benign conditions and PDAC patient groups. Statistica 13 (StatSoft, Tulsa, OK, USA), GraphPad Prism 6 (GraphPad Software, San Diego, CA, USA), and Analyse-it 4.0 (Analyse-it Software, Ltd., Leeds, UK) were used for statistical analysis.

## 3. Results

The biomarker selection strategy was described previously [11]. Customized electrochemiluminescent-based monoplex and multiplex immunoassays were subsequently developed using a MESO QuickPlex SQ 120 instrument. The cross-reactivity studies indicated that the degree of cross-reactivity across the immunoassays was generally <1%, based on the measurements in response to high concentrations of the recombinant proteins, either at the highest standard point (1st standard point, 2nd 4-plex) or 9× diluted the highest standard point (2nd–3rd standard point, 1st 4-plex) of the standard curve. Between 1.2 and 1.7% of nonspecific cross-reactions were observed between the CA-125 antigen and other capture antibodies (Table S1). It should be noted that nonspecific cross-reactivity was observed at recombinant protein concentrations that exceeded physiological levels, thereby reducing the chance of cross-reactivity in physiological human serum samples. By combining the U-PLEX-coupled capture antibodies and SULFO-TAG-conjugated detection antibodies used in the monoplex immunoassays, two 4-plex immunoassays of CA-125, HE4, KRT19 and FOLR1 (1st 4-plex) and CEA, HGF, OPG and Tie-2 (2nd 4-plex) were developed and evaluated. The calibration curves of CA-125, HE4, KRT19 and FOLR1 in the first 4-plex immunoassay or CEA, HGF, OPG and Tie-2 in the second 4-plex immunoassay were generated using the 4PL logistic regression models (Figure S1). The 4-plex immunoassay results correlated significantly with their respective monoplex immunoassay results (*p* < 0.00001), suggesting that the 4-plex immunoassays were comparable to the monoplex immunoassays for protein quantifications. Furthermore, there was a significant correlation of CA-125 protein measurements using the 4-plex immunoassay compared to Tosoh Bioscience AIA-2000 kit (*p* < 0.00001). The analytical performance of the two 4-plex immunoassays (Table 1 and Figure S2) showed recoveries of 96% to 111%, intra-assay precision of 2.2% to 10.6%, and inter-assay precision of 2.5% to 14%. The 4-plex immunoassays exhibited wide dynamic concentration ranges with the calibration curves covering 3–5 logs as defined by LLOD and ULOD.

The two 4-plex immunoassays were used to analyze the target protein levels in sera of 138 patients diagnosed with PDAC, 111 patients with benign pancreatic conditions, and 70 healthy controls. Among the 138 patients with PDAC, there were 56 with early-stage (IA/IB/IIA/IIB, 5/6/9/36; mean (SD) age, 65 (9) years; M/F, 20/36) and 82 with late-stage (III/IV, 19/63; 65 (10) years; 44/38) disease. Among 111 patients with benign pancreatic conditions, there were 53 with IPMN (63 (12) years; 20/33) and 58 with CP (51 (16) years; 42/16). Detailed clinicopathologic characteristics of the study cohort, including diagnosis, age, sex and anatomic stage, are shown in Table 2. The performance of the individual markers was compared to CA19-9 to discriminate between PDAC patients and benign conditions or healthy controls (Figure S3). Serum levels of HE4 and CA19-9 were significantly increased in benign conditions compared to healthy controls (HE4 at *p* < 0.0001 and CA19-9 at *p* < 0.05). Serum levels of CA-125, HE4, CEA, Tie-2 and CA19-9 were also significantly increased in PDAC patients compared to healthy controls (CA-125, HE4 and CA19-9 at *p* < 0.0001; CEA at *p* < 0.001; and Tie-2 at *p* < 0.01). Furthermore, serum levels of CA-125, HE4, KRT19, CEA and CA19-9 were significantly increased in PDAC patients compared to benign conditions (CA-125, CEA and CA19-9 at *p* < 0.0001; HE4 and KRT19 at *p* < 0.05).

**Figure 2:**
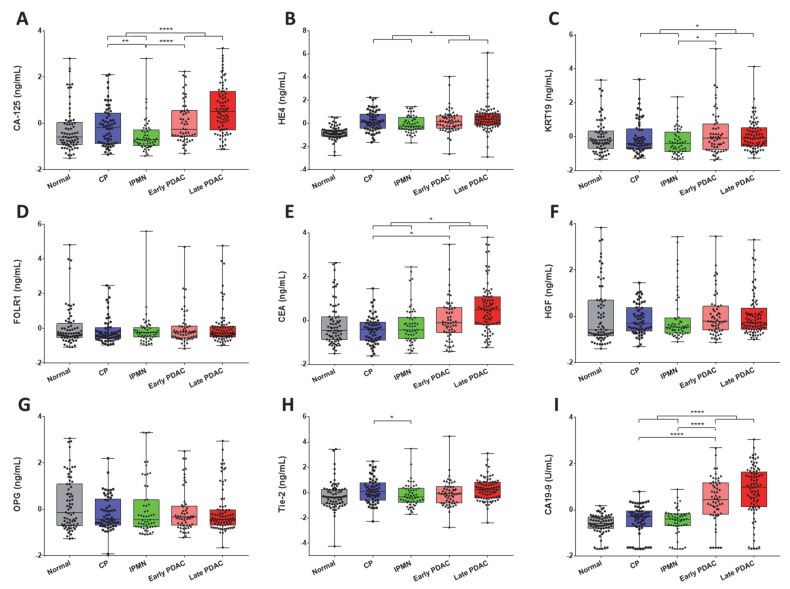
Analysis of biomarkers in sera from PDAC patients, benign conditions, and healthy controls. (**A**–**I**), CA-125, HE4, KRT19, FOLR1, CEA, HGF, OPG, Tie-2, and CA19-9 in PDAC patients, benign conditions, and healthy controls are demonstrated in overlaid scatterplots and boxplots. Only serum levels of biomarkers demonstrating significant differences between CP, IPMN, and early stage PDAC, (or benign and PDAC) are asterisked (Mann–Whitney U test). Biomarker data were transformed prior to analysis (log-transformation followed by z-score). Bars indicate median value. *, *p* < 0.05; **, *p* < 0.01; ****, *p* < 0.0001.

Serum levels of individual biomarkers were further assessed in different subgroups consisting of 70 healthy controls, 58 CP, 53 IPMN, 56 PDAC early-stage, and 82 PDAC late-stage patients (Table 3 and Figure 2). Serum levels of CA-125 and Tie-2 were significantly lower in IPMN compared with CP patients (CA-125 at *p* < 0.01 and Tie-2 at *p* < 0.05); however, there was no significant difference in serum levels of other biomarkers between IPMN and CP patients. Serum levels of CEA and CA19-9 were significantly increased in early stage PDAC compared to CP patients (CEA at *p* < 0.05 and CA19-9 at *p* < 0.0001). Serum levels of CA-125, KRT19 and CA19-9 were also significantly increased in early stage PDAC compared to IPMN patients (CA-125 and CA19-9 at *p* < 0.0001; KRT19 at *p* < 0.05).

**Figure 3:**
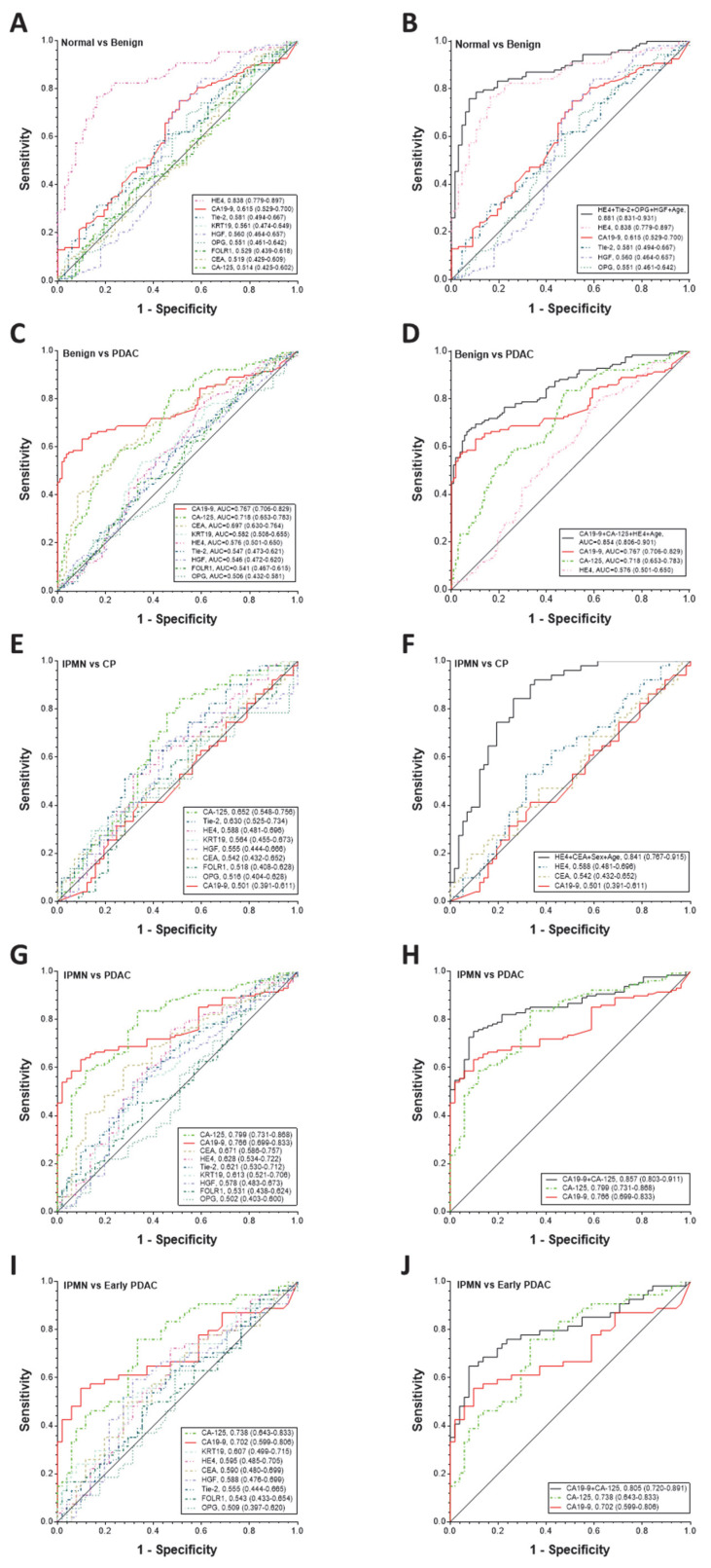
Diagnostic performance of individual or combination of serum biomarkers in detection of early stage PDAC. Diagnostic performance of CA19-9, CA-125, HE4, KRT19, FOLR1, CEA, HGF, OPG, and Tie-2 as individual markers (**A**,**C**,**E**,**G**,**I**) and their complementary (**B**,**D**,**F**,**H**,**J**) in differentiating patients with benign versus healthy controls (A&B) or PDAC versus benign (**C**,**D**) or IPMN versus CP (**E**,**F**) or PDAC versus IPMN (**G**,**H**) or early stage PDAC versus IPMN (**I**,**J**). ROC curves with AUCs are presented along with their 95% CI in brackets. Logistic regression modeling was constructed including sex and age as cofactors and backward stepwise selected z-score transformed variables with the highest performance. The four-marker panel of HE4, Tie-2, OPG and HGF including age as a cofactor with an AUC = 0.881 (0.831–0.931) for benign versus healthy controls, the three-marker panel of CA19-9, CA-125 and HE4 including age as a cofactor with an AUC = 0.854 (0.806–0.901) for PDAC versus benign, the two-marker panel of HE4 and CEA including sex and age as cofactors with an AUC = 0.841 (0.767–0.915) for IPMN versus CP, the two-marker panel of CA19-9 and CA-125 with an AUC = 0.857 (0.803–0.911) for PDAC versus IPMN, and the two-marker panel of CA19-9 and CA-125 with an AUC = 0.805 (0.720–0.891) for early stage PDAC versus IPMN significantly improved the individual biomarker performance (*p* value: <0.0001, 0.0004, <0.0001, 0.0005, or 0.0113 for CA19-9; Delong test).

Based on ROC curve analysis (Figure 3A,C,E,G,I), the three best biomarkers to separate benign from healthy controls were HE4 (0.838 (0.779–0.897)), CA19-9 (0.615 (0.529–0.700)), and Tie-2 (0.581 (0.494–0.667)). The three best biomarkers to separate PDAC from benign were CA19-9 (0.767 (0.706–0.829)), CA-125 (0.718 (0.653–0.783)), and CEA (0.697 (0.630–0.764)). The three best biomarkers to separate IPMN from CP were CA-125 (0.652 (0.548–0.756)), Tie-2 (0.630 (0.525–0.734)), and HE4 (0.588 (0.481–0.696)). The three best biomarkers to separate PDAC from IPMN were CA-125 (0.799 (0.731–0.868)), CA19-9 (0.766 (0.699–0.833)), and CEA (0.671 (0.586–0.757)). Furthermore, the three best biomarkers to separate early stage PDAC from IPMN were CA-125 (0.738 (0.643–0.833)), CA19-9 (0.702 (0.599–0.806)), and KRT19 (0.607 (0.499–0.715)).

Logistic regression modeling was constructed by backward stepwise selection using z-score transformed variables including sex and age as cofactors (Table 4 and Figure 3B,D,F,H,J). A four-marker panel of HE4 (*p* = 0.0001), Tie-2 (*p* = 0.0039), OPG (*p* = 0.0213), and HGF (*p* = 0.0009) including age (*p* = 0.0293) as a cofactor remained in the model, which had an AUC of 0.881 (0.831–0.931) that was greater than the individual biomarkers for benign versus healthy controls (*p* value: all including CA19-9 at <0.0001, except HE4 at 0.0287). In this four-marker panel, HE4 was a major contributor with an AUC of 0.838, which itself was significantly higher than CA19-9 (*p* < 0.0001). A three-marker panel of CA19-9 (*p* = 0.0000), CA-125 (*p* = 0.0090), and HE4 (*p* = 0.0092) including age (*p* = 0.0004) as a cofactor remained in the model that had an AUC of 0.854 (0.806–0.901), which was greater than the individual biomarkers for PDAC versus benign (*p* value: CA19-9 at 0.0004, CA-125 at 0.0001, and HE4 at <0.0001). A two-marker panel of HE4 (*p* = 0.0046) and CEA (*p* = 0.095) including sex (*p* < 0.0001) and age (*p* = 0.0002) as cofactors remained in the model that had an AUC of 0.841 (0.767–0.915), which was greater than the individual biomarkers for IPMN versus CP (*p* value: CA19-9, HE4 and CEA, all at <0.0001). A two-marker panel of CA19-9 (*p* = 0.0022) and CA-125 (*p* = 0.0088) remained in the model, which had an AUC of 0.857 (0.803–0.911) that was greater than the individual biomarkers for PDAC versus IPMN (*p* value: CA19-9 at 0.0005 and CA-125 at 0.0275). Furthermore, the same two-marker panel of CA19-9 (*p* = 0.0097) and CA-125 (*p* = 0.0277) remained in the model that had an AUC of 0.805 (0.720–0.891), which was greater than the individual biomarkers for early stage PDAC versus IPMN (*p* value: CA-125 at 0.0966 and CA19-9 at 0.0113).

As shown in Table 4, At a fixed SP of 80% and including sex and age as cofactors, the two-marker panel of HE4 and CEA significantly improved SN in separating IPMN from CP in comparison to that of CA19-9 alone (74% vs. 21%, *p* < 0.0001). At a fixed SP of 80%, the two-marker panel of CA19-9 and CA-125 significantly improved SN in detecting PDAC from IPMN in comparison to that of CA19-9 alone (78% vs. 41%, *p* < 0.0001). At the same SP of 80%, the same two-marker panel of CA19-9 and CA-125 also significantly improved SN in detecting early stage PDAC from IPMN in comparison to that of CA19-9 alone (72% vs. 59%, *p* = 0.0078).

## 4. Discussion

In this study, two 4-plex electrochemiluminescent-based immunoassays were developed with appropriate analytical performance for biomarker validation studies. They were applied to a set of patient sera to evaluate the performance of candidate biomarkers individually and in combination for their ability to complement CA19-9 for the early detection of PDAC. A three-marker panel of CA19-9, CA-125, and HE4 including age as a cofactor was firstly identified for detecting PDAC from benign conditions, including CP and IPMN. A two-marker panel of CA19-9 and CA-125 was further identified for detecting PDAC, as well as early stage PDAC from IPMN only. These panels showed strong diagnostic performance and significant improvement over the use of CA19-9 alone in terms of AUC. At a fixed SP of 80%, the panel of CA19-9 and CA-125 significantly improved SN in detecting PDAC (78% vs. 41%) as well as early stage PDAC (72% vs. 59%) from IPMN only, respectively, demonstrating that CA-125 was significantly complementary to CA19-9 in the detection of PDAC, as well as early stage PDAC from IPMN only.

Elevated CA-125 levels have been found in the sera of PDAC patients, and the utility of CA-125 as a biomarker in the management of PDAC patients has also been evaluated in several studies [13,14,15,16,17]. CA-125 was found to be superior to CA19-9 in predicting the resectability of PDAC [16], and postoperative serum CEA and CA-125 levels were shown to be supplementary to perioperative CA19-9 levels in predicting operative outcomes of PDAC [17]. The three-marker panel of CA-125, CA19-9 and LAMC2 was demonstrated to be able to significantly improve upon the performance of CA19-9 alone in discriminating early stage PDAC from benign conditions or CP [13]. In our study, compared with IPMN patients, the serum CA-125 levels of PDAC, as well as early stage PDAC, were significantly increased; in terms of AUC, CA-125 appeared to show better diagnostic performance than CA19-9, although not to a statistically significant degree.

While serum HE4 as a biomarker for ovarian cancer has been well recognized [18], serum HE4 levels were also found to be higher in cases with PDAC than in the controls with an SN of 45.83% and SP of 93.75% when the cutoff was set at 4.59 ng/mL [19], and the combination of HE4 and CA19-9 increased the SN to 83.33% [19,20]. Consistent with these reports, our study showed that the serum HE4 levels of benign conditions, as well as PDAC, were significantly increased compared with healthy controls; the serum HE4 level was also significantly increased in PDAC when compared with benign conditions. A four-marker panel of HE4, Tie-2, OPG, and HGF including age as a cofactor was also identified to distinguish benign conditions, including CP and IPMN, from healthy controls. HE4 was a major contributor in this panel and its performance in separating the two groups was significantly better than CA19-9, as well.

Tie-2 is a transmembrane tyrosine kinase receptor for angiopoietins and is crucial for angiogenesis and vascular maintenance. It has been reported that Foretinib simultaneously inhibited cancer cells and lymphatic endothelial cells to reduce pancreatic tumor growth in vivo, and suppressed angiogenesis and lymphangiogenesis by blocking VEGFR-2/3 and Tie-2 signaling [21]. We previously demonstrated that serum levels of Tie-2 were elevated in prostate cancer patients with higher Gleason scores [9,22]. In this study, compared with healthy controls, the serum Tie-2 level of PDAC patients was significantly increased, while the increase of the serum Tie-2 level in benign conditions was not statistically significant. The elevated levels of serum OPG have been found to be associated with poor prognosis in several cancer types [23,24]. The three-marker panel of CA19-9, ICAM-1 and OPG was identified to as highly discriminatory for PDAC versus healthy subjects demonstrating SNs ranging from 77–88% with 90% SP, and showed significant improvement over CA19-9 by ROC analysis [25]. The elevated level of HGF in cancer was reported to predict a more aggressive biology in breast, gastric, and pancreatic cancer patients [26,27]. Combining circulating tumor DNA and protein biomarker-based liquid biopsies demonstrated an increased SN of 64% in a blood test including plasma HGF for early stage PDAC [27].

Cyfra 21-1, a soluble fragment of KRT19, has already been shown to be a useful serum biomarker in lung, esophageal, breast, colorectal, ovarian, and pancreatic cancers [28,29,30,31,32]. In a prospective single-centre study, Cyfra 21-1 was shown to be an independent predictor for overall survival by multivariate analysis, and may serve as a novel, potent serum biomarker for monitoring treatment response and assessing prognosis in advanced PDAC [28]. In our study, compared with benign conditions, the serum KRT19 level of PDAC patients was significantly increased. Furthermore, the serum KRT19 level in early stage PDAC was also significantly increased when compared with IPMN patients. FOLR1 was reported to be significantly elevated in sera of ovarian cancer patients compared to both healthy controls and benign gynecological conditions and may be a new biomarker for ovarian cancer [33]; however, there were no studies that validated FOLR1 as a reliable serum biomarker for pancreatic cancer in a large cohort of patient samples.

CEA is a glycoprotein of cell surface whose levels are elevated in 30%–60% of patients with PDAC, but has relatively low SN and SP compared to CA19-9 [34,35]. In addition to CA19-9, it is also the most commonly used biomarker for the diagnosis and follow-up of IPMN. However, due to its low sensitivity (18%), CEA cannot be used as a screening method for malignant IPMNs, while it can be used to rule-in IPMN malignancy because of its high specificity (95%) [34]. In our study, the CEA serum level was found significantly increased in PDAC patients when compared with healthy controls or benign conditions; and the CEA serum level was also significantly increased in early stage PDAC when compared with CP.

IPMNs are epithelial mucin-producing cystic neoplasms that originated from the pancreatic ductal system. They have been characterized as one of three PDAC precursors, and the other two are pancreatic intraepithelial neoplasia (PanIN) and mucinous cystic neoplasia (MCN) [36]. IPMNs can be categorized macroscopically into three types: main duct (MD), branch duct (BD) and mixed type. The mode of duct involvement is related to risk assessment and guides therapy decision-making (i.e., resect versus follow-up) [36,37]. Approximately 25% of the pancreatic neoplasms resected surgically and 50% of pancreatic cysts detected incidentally are IPMNs, which can be benign or malignant in nature [38]. It is important to develop more useful serum biomarkers for the detection and surveillance of IPMN [11,39]. In this study, a two-marker panel of HE4 and CEA was shown to outperform CA19-9 in separating patients with IPMN from CP (SN: 74% vs. 21%), providing new insight into this field. However, in future studies, IPMNs need to be further characterized in terms of pathological features (i.e., the affected pancreatic ductal region, the level of epithelial dysplasia, and the number of masses) in order to draw clear conclusions.

In this study, two marker panels of CA19-9 and CA-125 or HE4 and CEA were demonstrated to be clinically useful to separate IPMN from PDAC as well as early stage PDAC or CP. These multivariate models will require additional validation. However, our results provide additional evidence of the utilities of these serum biomarkers and their complementary values as panels in the early detection of PDAC, which also serves as a cross-validation study via parallel analyses in a large, independent sample cohort in a multiplex immunoassay format. The development and application of multiple high-throughput screening technologies have made it possible that a large number of ‘-omic’ data, including genomics, epigenomics, transcriptomics, proteomics, and metabolomics, that may be combined as diagnostic or prognostic indices [40,41,42]. The selection of optimal panels through stepwise multivariate logistic regression allowed us to identify markers that are complementary in detecting specific disease conditions. However, for such panels to be used as an in vitro diagnostic multivariate index assay (IVDMIA), additional development work and large-scale multi-site independent validation studies will be required [43,44,45].

## 5. Conclusions

Two electrochemiluminescent-based multiplex immunoassays were developed, demonstrating appropriate analytical performance to evaluate serum biomarkers that may complement CA19-9 for early detection of PDAC. The biomarker panels identified in this study warrant additional clinical validation to determine their role in early detection of pancreatic cancer, which could lead to earlier intervention and better outcomes.

## Figures and Tables

**Table 1:** Analytical performances of two 4-plex immunoassays.

Marker Name		CA-125	HE4	KRT19	FOLR1	CEA	HGF	OPG	Tie-2
Quality control, Mean, pg/mL (Intra-/inter-assay CV%)	Sigma	1116.3 (4.4/11.1)	302.4 (3.8/2.5)	1103.1 (10.6/14.0)	333.0 (5.2/5.7)	260.0 (2.2/5.4)	240.2 (2.7/3.4)	217.4 (5.6/5.4)	6230.8 (3.2/3.5)
	Low	1873.7 (5.8/9.1)	83.0 (7.8/8.8)	726.7 (4.7/10.6)	393.4 (3.9/11.0)				
	High	84,223.9 (6.6/9.6)	1116.8 (7.9/9.6)	897.7 (9.3/6.0)	899.5 (4.3/8.2)				
%Recovery, mean (range)		96 (87–110)	99 (81–110)	102 (91–124)	101 (91–108)	101 (95–126)	111 (81–149)	103 (73–124)	100 (89–116)
HillSlope		0.9400	0.9800	1.1700	0.8700	0.9999	1.3771	1.1541	0.9974
R^2^		0.9999	0.9999	0.9999	0.9978	0.9998	0.9982	0.9926	1.0000
LLOD (pg/mL)		8.5	0.0	98.4	4.7	1.5	9.0	7.7	1.2
ULOD (pg/mL)		500,000.0	7500.0	37,500.0	175,000.0	50,000.0	12,000.0	100,000.0	40,000.0
4-plex vs. Monoplex, Pearson R *		0.9969	0.9924	0.9655	0.9668	0.999	0.9892	0.9599	0.9883
4-plex vs. Commercial kit, Pearson R ^#^		0.9715	ND	ND	ND	ND	ND	ND	ND

NOTE: First 4-plex assay includes CA-125, HE4, KRT19 & FOLR1. Second 4-plex includes CEA, HGF, OPG & Tie-2. LLOD, lower limit of detection. ULOD, upper limit of detection. Sigma and internal low & high QCs, diluted at either 4- or 8-fold. * 4-plex vs. monoplex in 30 patient sera. ^#^ 4-plex (pg/mL) vs. Tosoh Bioscience AIA-2000 (U/mL) in 30 patient sera. *p* < 0.00001 for all Pearson R. ND, not determined.

**Table 2:** Clinicopathologic characteristics of the study cohort.

Variables	Number (%)
Total	319
Healthy controls	70 (21.9)
Age (years)	
Mean ± SD	33 ± 14
Range (Median)	21–67 (27)
Gender	
Male	35 (50.0)
Female	35 (50.0)
Benign conditions	111 (34.8)
Age (years)	
Mean ± SD	57 ± 15
Range (Median)	13–84 (59)
Gender	
Male	62 (55.9)
Female	49 (44.1)
CP	58 (52.3)
IPMN	53 (47.7)
PDAC	138 (43.3)
Age (years)	
Mean ± SD	65 ± 10
Range (Median)	30–92 (65)
Gender	
Male	64 (46.4)
Female	74 (53.6)
Early stage	56 (40.6)
IA/IB/IIA/IIB	5/6/9/36
Late stage	82 (59.4)
III/IV	19/63

NOTE: CP, chronic pancreatitis. IPMN, intraductal papillary mucinous neoplasm.

**Table 3:** Statistics of individual biomarkers in healthy controls, benign conditions, and PDAC patients.

Biomarker	Subgroup	Number	Min	Max	Median	Mean	IQR
CA-125	Controls	70	1.7	505.9	5.8	29.9	9.0
	CP	58	2.1	201.4	9.8	23.0	18.2
	IPMN	52 ^a^	1.9	506.1	4.8	17.4	4.8
	Early stage	55 ^a^	2.2	244.7	8.9	29.6	19.8
	Late stage	79 ^a^	2.8	896.7	24.4	79.0	67.4
HE4	Controls	70	0.4	1.8	1.0	1.0	0.3
	CP	58	0.7	3.9	1.5	1.7	0.8
	IPMN	52 ^a^	0.7	2.7	1.3	1.5	0.6
	Early stage	56	0.4	8.6	1.5	1.8	0.7
	Late stage	80 ^a^	0.4	21.2	1.7	2.1	0.7
KRT19	Controls	67 ^a^	0.7	115.5	2.4	7.6	2.8
	CP	57 ^a^	0.7	119.1	1.9	6.6	3.2
	IPMN	51 ^a^	0.7	38.7	1.9	3.6	2.8
	Early stage	55 ^a^	0.7	866.5	2.7	24.0	5.5
	Late stage	76 ^a^	0.8	267.6	2.7	7.9	3.6
FOLR1	Controls	68 ^a^	0.7	174.4	1.5	7.6	1.3
	CP	57 ^a^	0.8	20.0	1.4	2.7	0.9
	IPMN	52 ^a^	0.8	360.5	1.6	8.7	0.8
	Early stage	55 ^a^	0.7	158.5	1.6	5.5	1.1
	Late stage	79 ^a^	0.8	165.4	1.6	6.2	1.0
CEA	Controls	70	0.4	55.0	1.3	4.9	2.0
	CP	58	0.3	13.3	1.4	1.9	1.3
	IPMN	53	0.4	44.0	1.3	3.9	1.8
	Early stage	56	0.4	152.8	2.0	6.3	3.3
	Late stage	82	0.5	224.3	4.1	14.9	6.6
HGF	Controls	70	1.2	57.5	2.2	6.3	3.8
	CP	58	1.3	9.8	2.7	3.4	2.3
	IPMN	53	1.5	43.0	2.4	5.2	1.2
	Early stage	55 ^a^	1.5	43.8	2.9	4.9	2.5
	Late stage	80 ^a^	1.6	38.8	2.7	4.8	2.0
OPG	Controls	70	0.3	367.8	2.0	25.2	14.1
	CP	58	0.1	90.7	1.3	4.9	4.1
	IPMN	53	0.4	552.8	1.2	38.2	4.1
	Early stage	54 ^a^	0.4	150.6	1.5	10.9	2.3
	Late stage	79 ^a^	0.2	300.4	1.3	12.7	1.6
Tie-2	Controls	70	14.2	182.1	51.6	58.1	20.3
	CP	58	27.3	131.9	59.9	65.0	26.5
	IPMN	53	33.0	183.8	51.5	57.4	18.8
	Early stage	55 ^a^	23.5	255.9	56.2	61.0	23.5
	Late stage	79 ^a^	26.2	162.0	63.5	65.9	25.9
CA19-9	Controls	70	<0.1	71.6	11.4	15.0	13.3
	CP	58	<0.1	203.2	20.3	31.3	33.1
	IPMN	53	<0.1	386.9	16.7	28.8	18.5
	Early stage	56	<0.1	27027.8	130.8	1169.7	725.1
	Late stage	82	<0.1	25110.7	354.8	1693.2	1569.7

NOTE: all biomarkers are at ng/mL, except CA19-9 at U/mL. IQR, interquartile range. ^a^ Missing data in 16 cases due to either insufficient sample (2 cases) or result outside the assay measuring range (14 cases). CP, chronic pancreatitis. IPMN, intraductal papillary mucinous neoplasm.

**Table 4:** Performance of individual and combined biomarkers for diagnosis of early stage PDAC.

	AUC (95% CI)	SN (%)	SP (%)
**IPMN vs. CP**			
CA19-9	0.501 (0.391–0.611)	21	80
HE4	0.588 (0.481–0.696)	25	80
CEA	0.542 (0.432–0.654)	27	80
HE4 + CEA + Sex + Age	0.841 (0.767–0.915)	74 *	80
**IPMN vs. PDAC**			
CA19-9	0.766 (0.699–0.833)	41	80
CA-125	0.799 (0.731–0.868)	66	80
CA19-9 + CA-125	0.857 (0.803–0.911)	78 ^@^	80
**IPMN vs. Early stage PDAC**			
CA19-9	0.702 (0.599–0.806)	59	80
CA-125	0.738 (0.643–0.833)	48	80
CA19-9 + CA-125	0.805 (0.720–0.891)	72 ^#^	80

Note: PDAC, pancreatic ductal adenocarcinoma. IPMN, intraductal papillary mucinous neoplasm. CP, chronic pancreatitis. AUC, area under curve. CI, confidence interval. SN, sensitivity. SP, specificity. One-tailed paired test comparing sensitivity against CA19-9: *, *p* < 0.0001; ^@^, *p* < 0.0001; ^#^, *p* = 0.0078.

## Supplementary Materials

The following are available online at https://www.mdpi.com/article/10.3390/biomedicines9121897/s1, Figure S1: Calibration curves of two 4-plex assays, Figure S2: Intra-assay and inter-assay variability of three pooled human sera with the known protein measurements (Sigma, internal Low and High QCs), Figure S3: Analysis of serum biomarkers of two 4-plex assays in PDAC patients, benign conditions, and healthy controls, Table S1: Assay specificity of two 4-plex immunoassays.

## Abbreviations

CA19-9: cancer antigen 19-9; CA-125, cancer antigen 125; HE4, human epididymis protein 4; KRT19, cytokeratin-19; FOLR1, folate receptor 1; CEA, carcinoembryonic antigen; HGF, hepatocyte growth factor; OPG, osteoprotegerin; Tie-2, TEK receptor tyrosine kinase.
