# Supplementary material for: Validation of Serum Biomarkers That Complement CA19-9 in Detecting Early Pancreatic Cancer Using Electrochemiluminescent-Based Multiplex Immunoassays
Source: Biomedicines. 2021 Dec 14;9(12):1897. doi: 10.3390/biomedicines9121897 (PMC8698985; doi:10.3390/biomedicines9121897)
Supplement: Supplementary file 1 [file biomedicines-09-01897-s001.zip › biomedicines-1469779-supplementary.pdf]

biomedicines

Supplement Materials

Table S1. Assay specificity of two 4-plex immunoassays. Percentage of cross-reactivity (single-antigen/single-detection antibody) was calculated based on fluorescence signals detected in response to high concentrations of the recombinant proteins either at the highest standard point (1st standard point, 2nd 4-plex) or 9x diluted the highest standard point (2nd-3rd standard point, 1st 4-plex) of the standard curve in single-antigen and single-detection antibody cross-reactivity studies (both with multiplexed capture antibodies).

**1st 4-plex**	**CA-125**	**HE4**	**KRT19**	**FOLR1**
**CA-125**		0.2/0.0	0.6/0.2	0.2/0.1
**HE4**	1.7/0.9		0.9/0.4	0.3/0.3
**KRT19**	1.2/0.7	0.2/0.1		0.2/0.1
**FOLR1**	1.5/0.8	0.2/0.1	0.8/0.3	

**2nd 4-plex**	**CEA**	**HGF**	**OPG**	**Tie-2**
**CEA**		0.0/0.0	0.2/0.0	0.0/0.0
**HGF**	0.0/0.0		0.2/0.3	0.0/0.0
**OPG**	0.0/0.0	0.1/2.4		0.0/0.1
**Tie-2**	0.0/0.0	0.0/0.0	0.2/0.1	

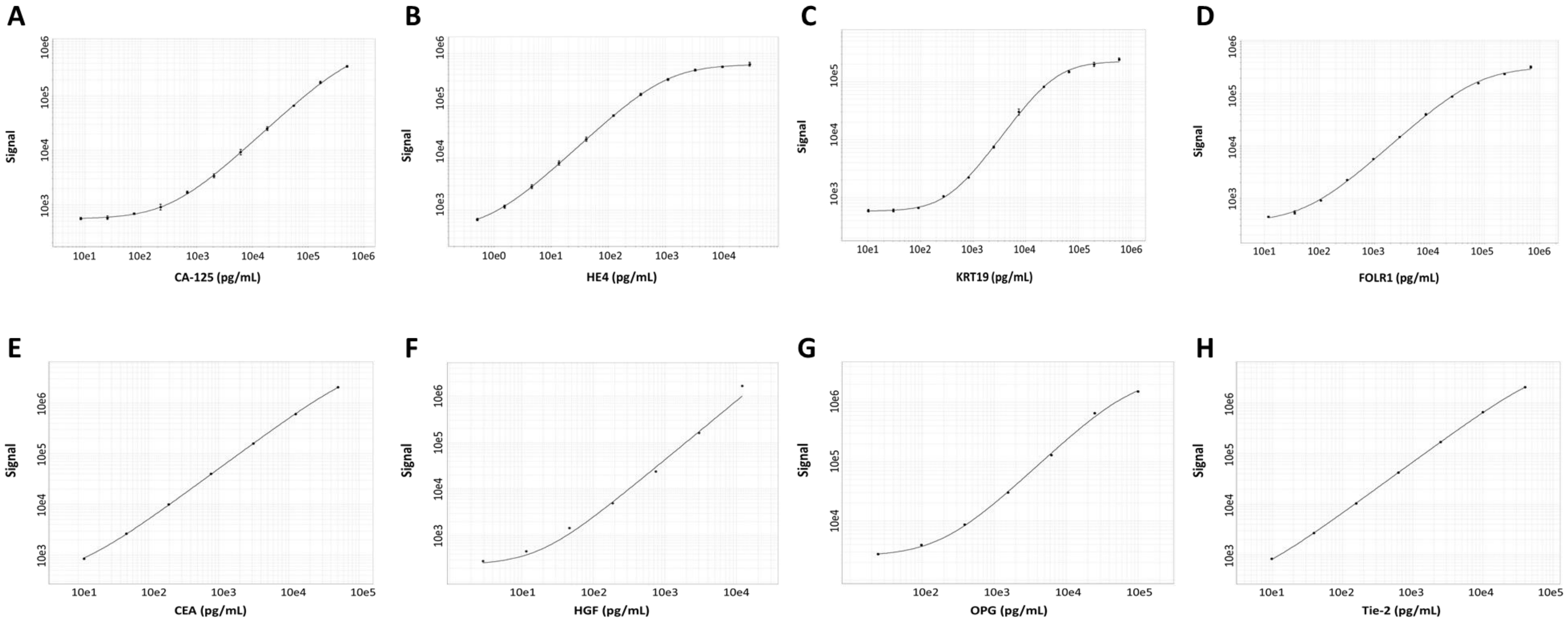

Figure S1. Calibration curves of two 4-plex assays. Calibration curves (**A-D**) of CA-125, HE4, KRT19 and FOLR1 in the first 4-plex immunoassay and calibration curves (**E-H**) of CEA, HGF, OPG and Tie-2 in the second 4-plex immunoassay were generated using the 4 parameter (4PL) logistic regression model.

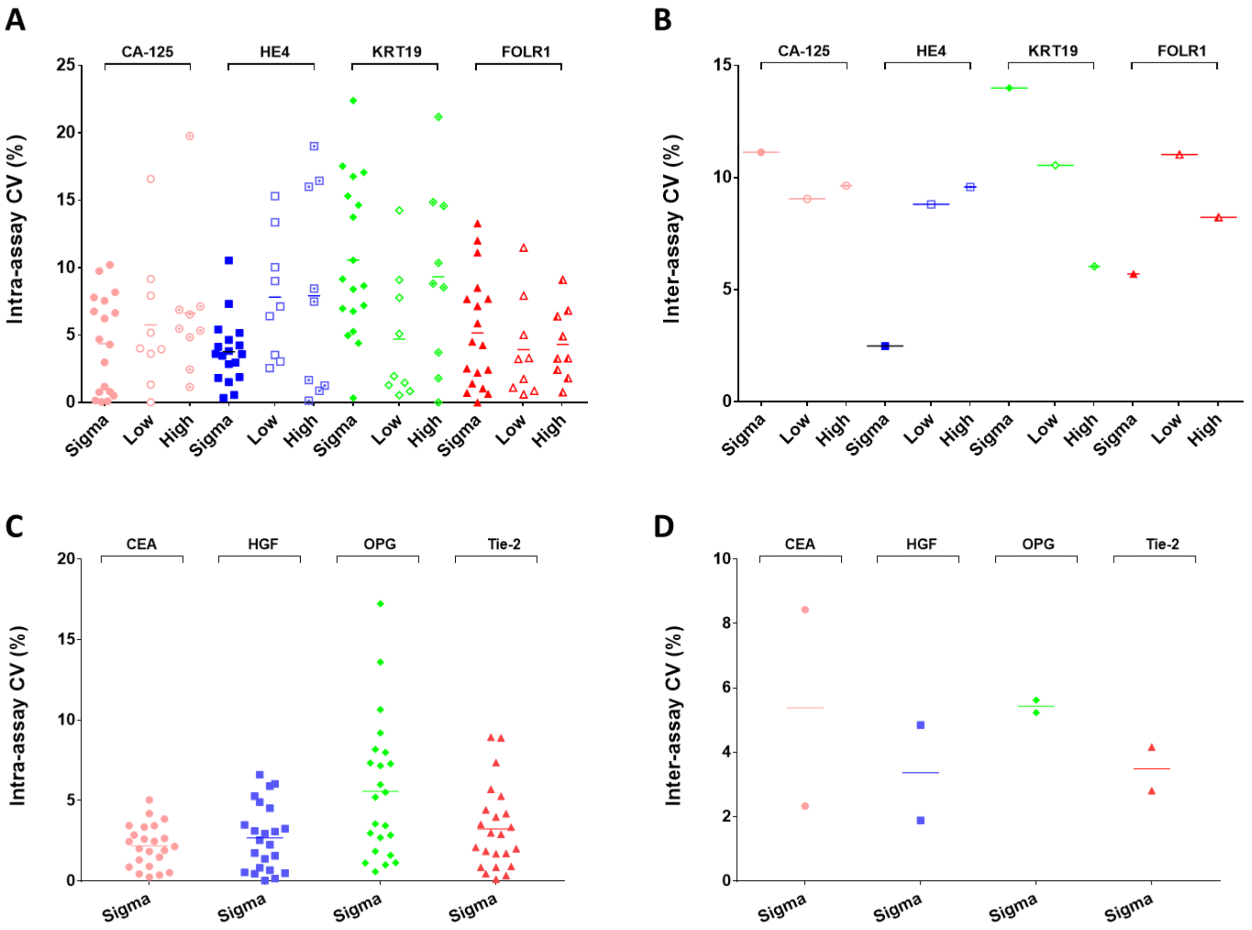

Figure S2. Intra-assay and inter-assay variability of three pooled human sera with the known protein measurements (Sigma, internal Low and High QCs). A, Intra-assay CVs of 1st 4plex assay were calculated from 36 Sigma, internal Low and High QCs in duplicate within a single assay. B, Inter-assay CVs of 1st 4plex assay were calculated from 7 (Sigma) or 9 (internal) independent assays on 2 different days, and each assay included at least 4 (Sigma) or 2 (internal) replicates. C, Intra-assay CVs of 2nd 4plex assay were calculated from 23 Sigma QCs in duplicate within a single assay. D, Inter-assay CVs of 2nd 4plex assay were calculated from 9 or 3 independent assays on 2 different days, and each assay included at least 4 or 2 replicates of Sigma QCs, respectively. Bars indicate mean value.

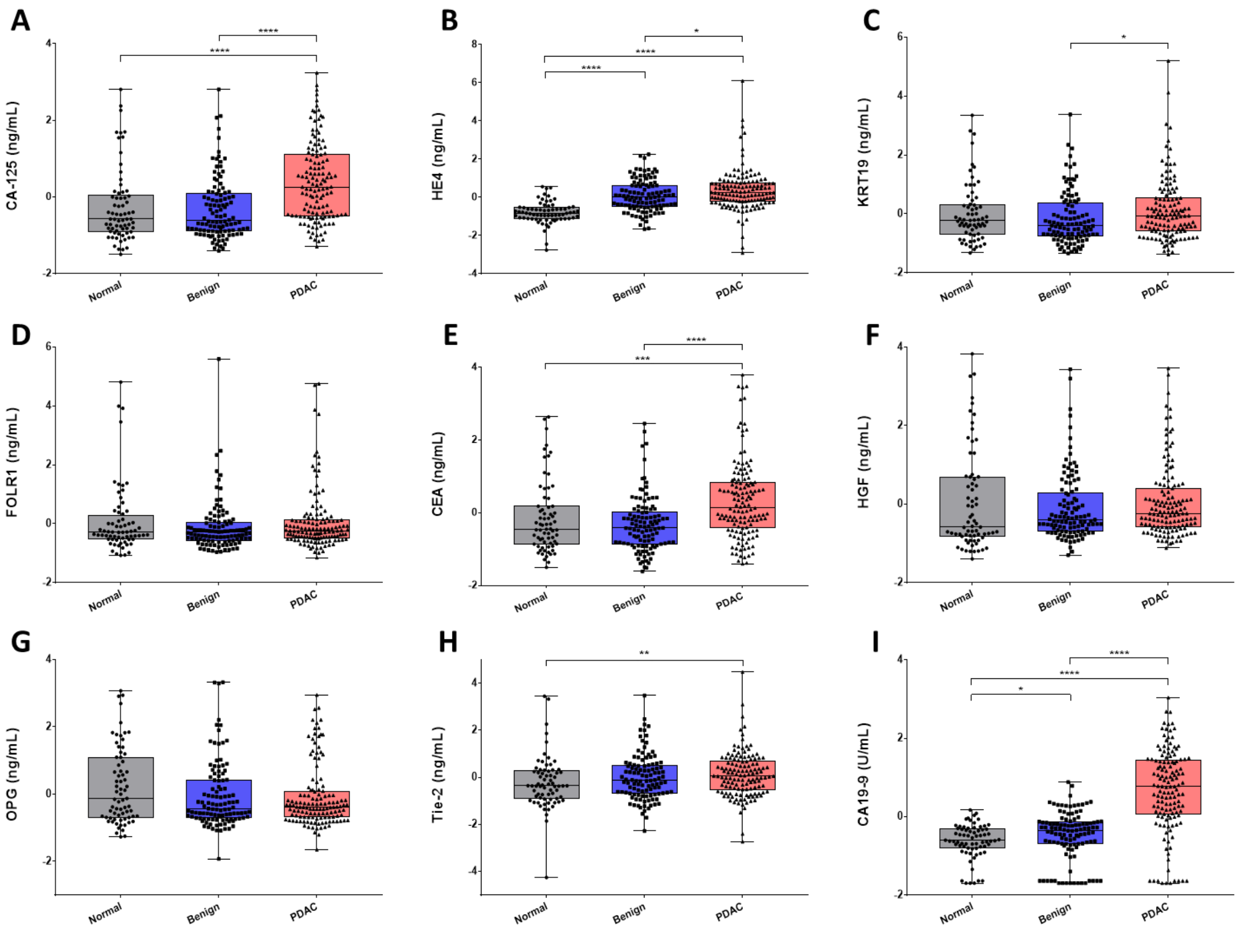

Figure S3. Analysis of serum biomarkers of two 4-plex assays in PDAC patients, benign conditions, and healthy controls. A-I, CA-125, HE4, KRT19, FOLR1, CEA, HGF, OPG, Tie-2 and CA19-9 in PDAC patients, benign conditions, and healthy controls are demonstrated in overlaid scatterplots and boxplots. Serum levels of biomarkers demonstrating significant differences between PDAC patients, benign conditions, and healthy controls are asterisked (Mann–Whitney U test). Biomarker data were transformed prior to analysis (log-transformation followed by z-score). Bars indicate median value. *, $p < 0.05$; **, $p < 0.01$; ***, $p < 0.001$; ****, $p < 0.0001$.
